# Control of DNA Damage Bypass by Ubiquitylation of PCNA

**DOI:** 10.3390/genes11020138

**Published:** 2020-01-29

**Authors:** Brittany M. Ripley, Melissa S. Gildenberg, M. Todd Washington

**Affiliations:** Department of Biochemistry, University of Iowa College of Medicine, Iowa City, IA 52242-1109, USA; brittany-ripley@uiowa.edu (B.M.R.); melissa-gildenberg@uiowa.edu (M.S.G.)

**Keywords:** DNA repair, DNA replication, Rad5, Rad18, translesion synthesis, template switching

## Abstract

DNA damage leads to genome instability by interfering with DNA replication. Cells possess several damage bypass pathways that mitigate the effects of DNA damage during replication. These pathways include translesion synthesis and template switching. These pathways are regulated largely through post-translational modifications of proliferating cell nuclear antigen (PCNA), an essential replication accessory factor. Mono-ubiquitylation of PCNA promotes translesion synthesis, and K63-linked poly-ubiquitylation promotes template switching. This article will discuss the mechanisms of how these post-translational modifications of PCNA control these bypass pathways from a structural and biochemical perspective. We will focus on the structure and function of the E3 ubiquitin ligases Rad18 and Rad5 that facilitate the mono-ubiquitylation and poly-ubiquitylation of PCNA, respectively. We conclude by reviewing alternative ideas about how these post-translational modifications of PCNA regulate the assembly of the multi-protein complexes that promote damage bypass pathways.

## 1. Introduction

Ionizing radiation, ultraviolet radiation, and a wide range of chemical agents damage DNA [1]. The resulting DNA lesions are problematic because, if unrepaired, they can lead to mutations and chromosomal re-arrangements. This genome instability, in turn, can cause cancers and a variety of other diseases [2,3,4,5]. These damage-induced mutations and chromosomal re-arrangements occur largely because DNA lesions interfere with normal DNA replication. DNA lesions in the template strand block the progress of classical DNA polymerases, those involved in normal DNA replication and repair. Thus, without a means of overcoming these replication blocks, DNA replication will not be completed, and cells will die.

Cells have evolved multiple DNA damage bypass pathways that mitigate the effects of DNA damage during replication [1,6,7]. These pathways include translesion synthesis, template switching, homologous recombination, and repriming. In this article, we focus on translesion synthesis and template switching because these two pathways are regulated largely through post-translational modifications of proliferating cell nuclear antigen (PCNA), an essential replication accessory factor [8]. Mono-ubiquitylation of PCNA, for example, promotes translesion synthesis. K63-linked poly-ubiquitylation of PCNA, by contrast, promotes template switching. 

An underappreciated aspect of the regulation of these DNA damage bypass pathways is the role played by the E3 ubiquitin ligases that facilitate the mono-ubiquitylation and poly-ubiquitylation of PCNA. A substantial amount of evidence suggests that the Rad18 protein, which is the E3 ubiquitin ligase responsible for PCNA mono-ubiquitylation [8], and the Rad5 protein, which is the E3 ubiquitin ligase responsible for PCNA poly-ubiquitylation [8], play a more direct role in orchestrating the events leading to DNA damage bypass than was originally thought. 

In this article, we will discuss the regulation of these DNA damage bypass pathways by ubiquitin modifications of PCNA. We will focus on the structure, function, and interactions of the E3 ubiquitin ligases Rad18 and Rad5 that are responsible for these PCNA modifications. Then, we will discuss alternative ideas about how ubiquitin modifications regulate the assembly of the multi-protein complexes that carry out DNA damage bypass. 

## 2. DNA Damage Bypass and PCNA Modifications

**Figure 1:**
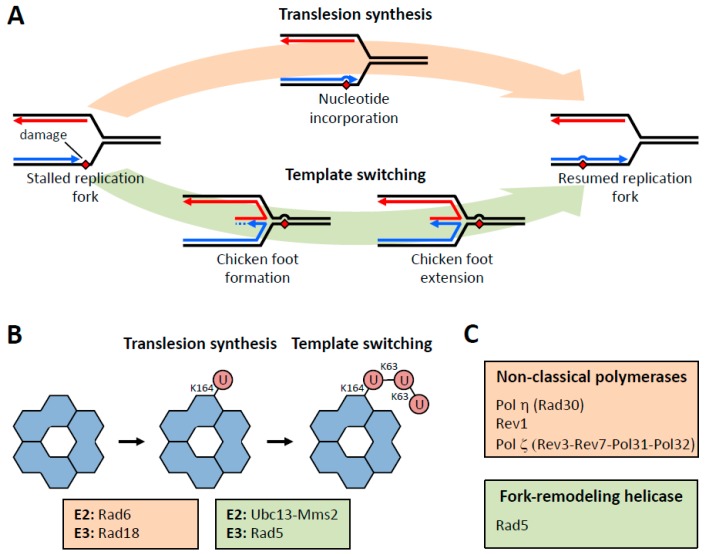
DNA damage bypass pathways and proliferating cell nuclear antigen (PCNA) modifications. (**A**) The translesion synthesis pathway involves nucleotide incorporation using the damaged DNA as a template. The template switching pathway involves the formation and extension of the chicken foot intermediate. (**B**) Mono-ubiquitylation of PCNA by Rad6–Rad18 promotes translesion synthesis, and poly-ubiquitylation of PCNA by Ubc13–Mms2–Rad5 promotes template switching. (**C**) Translesion synthesis involves non-canonical polymerases such as pol η, pol ζ, and Rev1.

The bypass of damaged DNA is carried out by two pathways: translesion synthesis and template switching (Figure 1). In translesion synthesis, the stalled classical DNA polymerase is replaced by one or more specialized, non-canonical DNA polymerases [6,7,9,10,11,12,13,14,15,16,17,18,19,20]. Each of these non-canonical DNA polymerases has evolved to efficiently incorporate nucleotides opposite one or more damaged DNA templates. For example, DNA polymerase (pol) η (Rad30) efficiently replicates through template thymine dimers and 8-oxoguanines [21,22]. Similarly, Rev1 efficiently replicates through minor-groove adducted guanines, exocyclic guanine lesions, and abasic sites [23,24,25]. This pathway can be error-prone (i.e., mutagenic) or error-free (i.e., non-mutagenic) depending on which non-canonical polymerase is utilized to bypass the lesion. 

In template switching, the stalled classical polymerase is replaced by a fork-remodeling helicase [6,7,26,27,28,29]. In yeast, this fork-remodeling helicase is Rad5. Although the precise role of Rad5 in template switching remains unclear, there is strong in vitro evidence showing that Rad5 uses the energy derived from ATP hydrolysis to reverse the replication fork [29]. This leads to the stalled strand base-pairing with the newly synthesized strand on the sister duplex to form the “chicken foot” intermediate (Figure 1) [29]. A DNA polymerase then extends the “middle toe” of the chicken foot structure, and Rad5 finally remodels the chicken foot structure back to a replication fork. This results in the damage having been bypassed, and normal DNA replication can resume. It is important to note, however, that the fork reversal activity of Rad5 has not been confirmed in wild-type cells.

These DNA damage bypass pathways are largely regulated by the ubiquitylation of PCNA (Figure 1) [8,30,31,32,33,34,35,36,37,38,39,40]. The mono-ubiquitylation of PCNA on lysine-164 promotes translesion synthesis [8]. This is catalyzed by the E2 protein Rad6 and the E3 protein Rad18. This is thought to trigger the recruitment and activation of effector proteins such as the translesion synthesis polymerases pol η, pol ζ (Rev3–Rev7–Pol31–Pol32), and the Rev1 protein. 

The K63-linked poly-ubiquitylation of PCNA on lysine-164 promotes template switching [8]. This modification is catalyzed by the E2 protein Ubc13 with the E2-like protein Mms2 and the E3 protein Rad5. Unlike mono-ubiquitylation, poly-ubiquitylation does not trigger the recruitment of an additional effector protein. This is because Rad5 is a dual-functional enzyme, being also the fork-remodeling helicase that forms the chicken foot intermediate [29].

## 3. Rad6–Rad18

**Figure 2:**
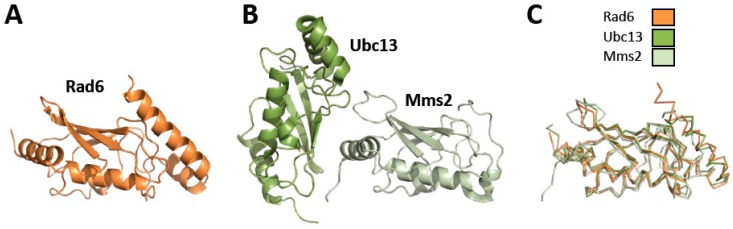
Structures of Rad6, Ubc13, and Mms2. (**A**) Ribbon diagram of Rad6 (orange) (1AYZ.pdb) [41]. (**B**) Ribbon diagram of Ubc13-Mms2 (dark green and light green, respectively) (1JAT.pdb) [43]. (**C**). Overlay of the structures of Rad6 (orange), Ubc13 (dark green), and Mms2 (light green).

**Figure 3:**
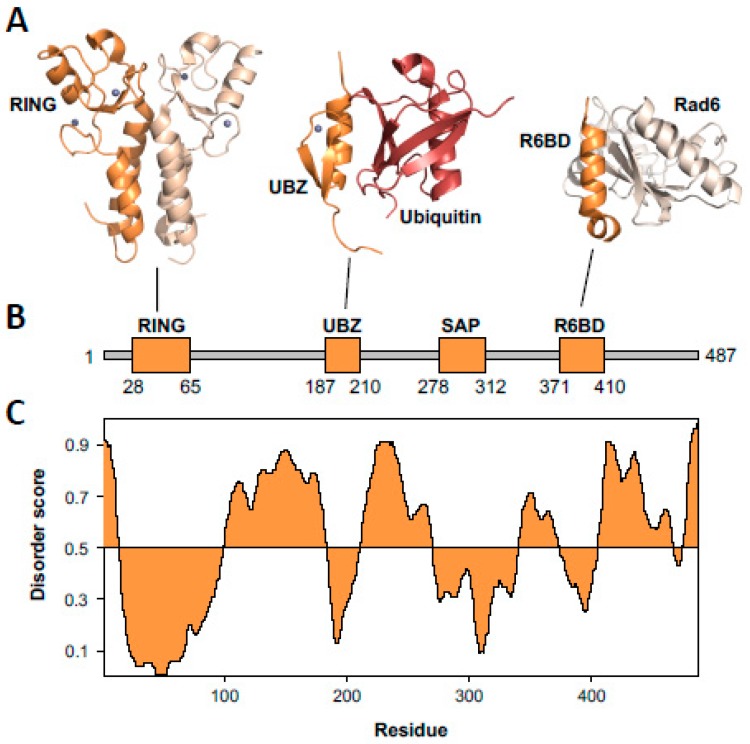
Structure of Rad18. (**A**) Ribbon diagrams of the Rad18 Really Interesting New Gene (RING) domain dimer (dark orange and light orange) (2Y43.pdb) [44], the Rad18 Ubiquitin-Binding, Zinc-Binding (UBZ) domain (dark orange) bound to ubiquitin (red) (2MRE.pdb) [45], and the Rad18 R6BD domain (dark orange) bound to Rad6 (light orange) (2YBF.pdb) [42]. The zinc ions are shown as spheres. (**B**) A linear diagram of Rad18 shows the structured domains as thick, orange rectangles and the intrinsically disordered regions as thin, grey rectangles. (**C**) A disorder plot of Rad18 was generated using the Protein Disorder Prediction Server [46].

Rad6–Rad18 is the E2 ubiquitin-conjugating enzyme/E3 ubiquitin ligase that catalyzes the mono-ubiquitylation of PCNA [8]. Rad6 is a 20 kDa globular protein. The X-ray crystal structures of both yeast and human Rad6 have been determined (Figure 2) [41,42]. Both yeast and human Rad6 proteins have a nearly identical fold to Ubc13, the E2 ubiquitin-conjugating enzyme that catalyzes the poly-ubiquitylation of PCNA, and to Mms2 [8]. Rad18 is a 55 kDa protein with several structured domains separated by intrinsically disordered regions (Figure 3). 

Unlike some E2:E3 pairs, which interact transiently and are often found not bound together, Rad6–Rad18 form a tight complex comprised of two Rad18 molecules and one Rad6 molecule [47]. While Rad6 is stable alone and is present in cells in high copy number, Rad18 is not found in the absence of Rad6 and is approximately 10 times less abundant in cells than Rad6 [48,49].

### 3.1. Structure and Function of Rad18

Structural studies of Rad18 have been limited to its structured domains and motifs. The structured domains include the Really Interesting New Gene (RING) domain, Ubiquitin-Binding, Zinc-Binding (UBZ) domain, SAF-A/B, Acinus, Pias (SAP) domain, and Rad6-Binding Domain (R6BD) (Figure 3). The regions of Rad18 between these structured domains are intrinsically disordered [46,50]. Intrinsically disordered regions are often sites for protein–protein interactions [51,52]. In Rad18, two such interaction motifs have been identified: a SUMO-interaction motif (SIM) and a pol η-binding region.

#### 3.1.1. The RING Domain

The X-ray crystal structure of the human Rad18 RING domain shows that this domain forms a homodimer (Figure 3) [44]. The RING domain (corresponding to residues 28 to 65 of yeast Rad18) is located at the N-terminus of Rad18. This is a C_3_HC_4_ zinc finger comprised of seven cysteine residues (corresponding to residues 28, 31, 43, 48, 51, 62, and 65 of yeast Rad18) and one histidine residue (corresponding to residue 45 of yeast Rad18) that coordinate two zinc ions. Two α-helicases (corresponding to residues 15 to 25 and residues 78 to 94 of yeast Rad18) flank the zinc finger. These α-helicases form a four-helix bundle in the RING domain homodimer. This four-helix bundle and a series of hydrogen bonds between residues of the zinc finger motifs stabilize the RING domain dimer. While RING domain dimerization is required for the function of several other RING E3 ubiquitin ligases, it is not known if this dimerization is required for the ligase function of Rad18.

The RING domain of Rad18 binds to Rad6 via a characteristic RING-E2 interface consisting of both hydrophobic and electrostatic interactions [44]. The RING domain binds Rad6 with a dissociation constant in the low micromolar range, and mutations in the RING domain that block this interaction greatly reduce Rad18’s ubiquitin ligase activity [44]. 

#### 3.1.2. The UBZ Domain

The NMR structure of the human Rad18 UBZ domain (corresponding to residues 187 to 210 of yeast Rad18) shows that this domain has an overall β-strand/β-strand/α-helix fold (Figure 3) [45]. It is a C_2_HC zinc-binding domain comprised of three cysteine residues (corresponding to residues 190, 193, and 210 of yeast Rad18) and one histidine residue (corresponding to residue 206 of yeast Rad18). NMR titrations show that the Rad18 UBZ domain binds to ubiquitin with a dissociation constant in the low micromolar range [45]. Moreover, the α-helix of the Rad18 UBZ (corresponding to residues 200 to 211 of yeast Rad18) and the β-strand 1 (corresponding to residues 187 to 190 of yeast Rad18) interact with the canonical hydrophobic patch of ubiquitin comprised of leucine-8, isoleucine-44, and valine-70. 

Interestingly, mutations in the UBZ domain do not block the ability of Rad18 to function as a ubiquitin ligase during the mono-ubiquitylation of PCNA (47). This suggests that this domain may be involved in a function of Rad18 other than translesion synthesis. In fact, the UBZ domain is essential for Rad18 auto-ubiquitylation [53], which localizes Rad18 to the cytoplasm making it unavailable to ubiquitylate nuclear proteins such as PCNA and Replication Factor C (RFC), the protein that loads PCNA onto DNA.

#### 3.1.3. The SAP Domain

This small domain (residues 278 to 312) is named after the three proteins in which it was first identified: SAF-A/B, Acinus, and Pias [54]. The structure of the Rad18 SAP domain has not yet been determined, but homology models predict that it has a helix–turn–helix fold similar to that of other SAP domains. This domain binds both single- and double-stranded DNA with dissociation constants in the low micromolar range [55]. It is not known, however, whether these DNA binding activities are necessary for Rad18 ligase function. Inactivation of the SAP domain does, however, affect the recruitment of non-canonical pol η to sites of DNA damage, indicating that this domain plays an important but poorly understood role in the recruitment of non-canonical DNA polymerases [53].

#### 3.1.4. The Rad6-Binding Domain

The R6BD is a small domain (residues 371 to 410) near the C-terminus of Rad18 that binds Rad6. This domain has a helix–loop–helix fold (Figure 3) [42]. This domain binds to the opposite side of Rad6 from the site of ubiquitin conjugation (cysteine-88) and interacts with β-strand 1, β-strand 2, and β-strand 3 of Rad6 [42]. Both surface plasmon resonance and NMR titrations show that the dissociation constant for R6BD and Rad6 is in the mid micromolar range [42]. Mutations in the R6BD in human Rad18 decrease the binding affinity of Rad18 for Rad6 and substantially reduce Rad18’s ubiquitin ligase activity. While the RING domain and the R6BD of Rad18 both bind Rad6, the functional significance of this bidentate interaction is unclear. One possibility is that, like other bidentate E2–E3 interactions, the RING domain can transiently dissociate from E2 facilitating the re-charging of E2 by E1 without the complex dissociating [56]. This could allow for the processive ubiquitylation of the PCNA subunits by Rad6–Rad18. 

#### 3.1.5. The SUMO-Interacting Motif

Yeast Rad18 contains a SIM (residues 139 to 142) in the predicted disordered region between the RING domain and the UBZ domain [57]. Conserved hydrophobic residues in the yeast SIM (leucine-139, isoleucine-141, and valine-142) are necessary for interaction with PCNA. Moreover, a fraction of PCNA during normal DNA replication is sumoylated, and the sumoylation of PCNA stimulates the ubiquitin ligase activity of Rad18. These findings support the notion that Rad18 is a SUMO-directed ubiquitin ligase [57]. 

#### 3.1.6. The Pol η-Binding Region

Pol η is a non-canonical DNA polymerase that carries out translesion synthesis through thymine–thymine dimers and 8-oxoguanine lesions [21,22]. Human pol η binds to a region in the disordered C-terminus of human Rad18 following the R6BD [55]. This interaction is dependent on phosphorylation of serine-409 of human Rad18 [58]. The lack of phosphorylation at this site results in an increased sensitivity to ultraviolet radiation and a decrease in nuclear foci containing pol η.

### 3.2. Other Interactions of Rad18

As described above, Rad18 interacts with Rad6, pol η, and PCNA. In addition to these proteins, Rad18 interacts with several other proteins involved in DNA damage bypass including RPA (replication protein A), Rev1, and Rad5.

#### 3.2.1. Replication Protein A

Replication Protein A (RPA) is a single-stranded DNA-binding protein involved in DNA replication, repair, and recombination [59]. It has three subunits: Rfa1, Rfa2, and Rfa3. The interaction between yeast RPA and Rad18 involves the Rfa1 and Rfa2 subunits [60]. This interaction is disrupted by the deletion of residues 112 to 192 of Rad18, which contain the SIM [60]. It is unclear, however, whether this is due to the loss of a direct interaction with RPA or of an indirect interaction with RPA through sumoylated PCNA or some other mediator protein.

#### 3.2.2. Rev1

Rev1 has two functions in DNA damage bypass. First, it is a non-canonical DNA polymerase that catalyzes translesion synthesis of minor-groove and exocyclic guanine adducts as well abasic sites [23,24,25]. Second, it is a structural protein that can simultaneously bind PCNA and other non-canonical DNA polymerases forming a Rev1 bridge [61,62]. In this way, it can help organize the structure of the multi-protein complexes that carry out DNA damage bypass. Rad18 and Rev1 have been shown to physically interact [63]. Despite this, however, the structural basis and functional implications of this interaction remain poorly understood.

#### 3.2.3. Rad5

Rad5 is the E3 ubiquitin ligase that is necessary for the template-switching pathway of DNA damage bypass (see below). Rad18 and Rad5 interact with each other [64]. This interaction has been partially mapped to residues 83 to 246 of Rad18, which contain both the SIM and the UBZ domain [64]. More work is needed to understand the structural basis and functional implications of this interaction.

## 4. Ubc13–Mms2–Rad5 

Ubc13–Mms2–Rad5 is the E2 ubiquitin-conjugating enzyme/E3 ubiquitin ligase that catalyzes the K63-linked poly-ubiquitylation of PCNA [8]. Ubc13 is an 18 kDa globular protein and is the actual E2 ubiquitin-conjugating enzyme. It forms a heterodimer with Mms2, a 16 kDa globular protein that is a ubiquitin-conjugating enzyme variant. This heterodimer is required for DNA damage bypass by template switching. The X-ray crystal structures of yeast and human Ubc13–Mms2 have been determined with and without ubiquitin covalently attached to Ubc13 (Figure 2) [43,65]. The structure of Ubc13 is similar to that of other E2 proteins, such as Rad6. Mms2 has a similar fold with slight modifications allowing it to form a binding surface for Ubc13.

**Figure 4:**
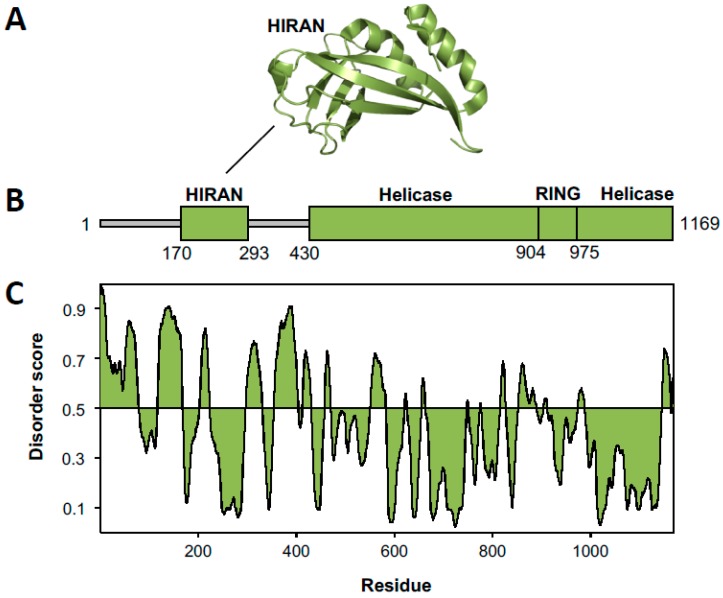
Structure of Rad5. (**A**) Ribbon diagram of the Rad5 HIP116 Rad5p N-terminal (HIRAN) domain (green) (4XZG.pdb) [68]. (**B**) A linear diagram of Rad5 shows the structured domains as thick, green rectangles and the intrinsically disordered regions as thin, grey rectangles. (**C**) A disorder plot of Rad5 was generated using the Protein Disorder Prediction Server [46].

Yeast Rad5 is a 134 kDa protein that has two functions [66,67]. First, it is an E3 ubiquitin ligase that binds Ubc13–Mms2 and forms K63-linked poly-ubiquitin chains on mono-ubiquitylated PCNA [8]. Second, it is a fork-remodeling helicase that converts stalled replication forks to chicken foot intermediates [29]. Like Rad18, Rad5 has structured domains separated by intrinsically disordered regions (Figure 4). Unlike Rad18, however, Rad5 can be overexpressed without Ubc13–Mms2.

### 4.1. Structure and Function of Rad5

Currently, there is no high-resolution structural information about Rad5. Our limited structural understanding of Rad5 has been obtained from homology modeling of its structured domains. These domains include the HIP116 Rad5p N-terminal (HIRAN) domain, the helicase domain, and the RING domain.

#### 4.1.1. HIRAN Domain

The HIRAN domain of Rad5 (corresponding to residues 170 to 293 of yeast Rad5) is located near the N-terminus of the protein and is flanked by intrinsically disordered regions. While there are no high-resolution structures of this domain, the HIRAN domain of Helicase-Like Transcription Factor (HLTF), a human homolog of Rad5, has been determined (Figure 4) [68,69,70]. The HIRAN domain contains an oligosaccharide/oligonucleotide-binding (OB) fold that binds the 3′ ends of DNA. Recognition of the 3′ OH is important for HIRAN binding, and the structure of the HIRAN domain prevents binding of double-stranded DNA. This suggests that the 3′ end of a DNA strand must be unwound before the HIRAN domain can bind it. The HIRAN domains of Rad5 and of HLTF are important for fork-remodeling activity. It has been proposed that the HIRAN domain is important for substrate recognition and positioning of the helicase domain on the DNA substrate [71].

The HIRAN domain of HLTF is required for unwinding forked DNA substrates containing double-stranded DNA on both the leading and the lagging strand of the fork without single-stranded gaps at the fork junction [71]. By contrast, this domain is not as important when unwinding forked DNA substrates with single-stranded gaps on either the leading or the lagging strand. However, unwinding of substrates with gaps on the lagging strand was more hindered by the deletion of the HIRAN domain than by substrates with gaps on the leading strand. This suggests that a functional HIRAN domain is more important when the 3′ end of the leading strand is located immediately at the fork junction [71]. 

#### 4.1.2. Helicase Domain

Rad5 is a member of the Swi/Snf subfamily within the SF2 superfamily of helicases [72,73]. The helicase domain of Rad5 (which begins around residue 430 of yeast Rad5 and ends at residue 1169) spans the C-terminal half of the protein. Like other helicases in this superfamily, the Rad5 helicase domain contains seven conserved motifs, including the Walker A and Walker B ATP-binding motifs. In the structures of other SF2 helicases, these conserved motifs constitute an ATP-binding site between the two RecA-like folds that make up the helicase domain [73]. Moreover, the helicase domain also binds the DNA substrate. 

The helicase domain is largely responsible for the ATPase and fork-remodeling activity of Rad5 [29]. The ATPase activity of Rad5 is stimulated by single-stranded DNA as well as double-stranded DNA in the context of three-way junctions (forks) and four-way junctions (chicken foot structures and Holiday junctions). In addition, Rad5 can unwind forked substrates with homologous arms (i.e., ones in which the primer strands of the leading and lagging arms are complementary to one another) but not heterologous arms [29]. Moreover, Rad5 is capable of regressing several hundred base pairs of DNA. Whether this is the result of processive or distributive unwinding is unclear.

In the case of human HLTF, footprinting experiments suggest that translocation occurs in the 3′–5′ direction, with the helicase domain positioned on the lagging strand template of the parental duplex in front of the fork [71]. For Rad5, ATP binding to the helicase domain induces the unwinding of the primer strand of the leading arm from the template strand, creating a free 3′ end [74]. This free 3′ end can then capture the primer strand of the lagging arm to form a four-way junction—a chicken foot structure or a Holiday junction. Formation of this four-way junction has been proposed to be the rate-limiting step of fork reversal [74]. 

#### 4.1.3. RING Domain

Swi/Snf helicases often contain a small, folded domain between the two RecA-like folds of the helicase domain [73]. In the case of Rad5, there is a RING domain (residues 904 to 975) inserted at this position. Homology models based on X-ray crystal structures of other RING domains show that this is a C_3_HC_4_ zinc finger-type domain comprised of seven cysteine residues (residues 914, 917, 932, 937, 940, 957, and 960) and one histidine residue (residue 934) that coordinate zinc ions [75]. There is no evidence of the RING domain of Rad5 forming dimers. Instead, the RING domain binds Ubc13 and is important for Rad5’s E3 ubiquitin ligase activity [64]. 

### 4.2. Structural Models of Full-Length Rad5

Though Rad5 has several structured domains, approximately 30% of it is likely unstructured. There are two large intrinsically disordered regions: one at the N-terminus (starting at residue 1 and ending at residue 169 in yeast Rad5), and one between the HIRAN domain and the helicase domain (starting at residue 294 and ending around residue 525 in yeast Rad5). The role of these unstructured regions is likely to provide conformational flexibility to Rad5, allowing the HIRAN domain and the helicase domain to reorient themselves with respect to one another. In addition, these regions likely mediate protein–protein interactions. 

A recent structural model of full-length Rad5 has been proposed on the basis of a combination of molecular simulations and small-angle X-ray scattering (SAXS) data [75]. The molecular simulation generated an ensemble of 5000 structures, which collectively agree well with the SAXS data. In this structural model, the disordered regions are highly flexible and adopt many conformational states. Interestingly, this structural model revealed the presence of an intra-molecular interaction between the HIRAN and the helicase domains, which resulted in a more compact structure than what would have been expected given the high degree of intrinsic disorder in the protein [75]. It should be noted, however, that the SAXS data only examined the protein in the absence of ATP, DNA, and other binding partners. Thus, it is unclear whether the putative HIRAN domain–helicase domain interaction persists in the presence of these ligands. 

### 4.3. Other Interactions of Rad5

As described above, Rad5 interacts with Ubc13 and Mms2. In addition to these proteins, Rad5 interacts with several other proteins including PCNA, Rev1, and Rad18.

#### 4.3.1. PCNA

Rad5 binds PCNA through its N terminus (amino acids 1–430) [8]. The precise residues involved in the interaction are unknown. This region contains a putative PCNA-interacting protein PIP-like motif (residues 6 to 13), which binds Rev1 (see below) [31,76]. It is not known if this motif mediates interactions with PCNA as well. Rad5 binds unmodified PCNA and mono-ubiquitylated PCNA with similar affinities [77]. 

#### 4.3.2. Rev1

Like Rad18, Rad5 interacts with Rev1, the non-canonical polymerase that helps to organize the structure of the complex that carries out DNA damage bypass [76]. Based on this, it has been suggested that Rad5 may regulate Rev1-mediated translesion synthesis. The structural basis of this interaction is known. An X-ray crystal structure shows that the Rev1 C-terminal domain, which binds PIP-like motifs, binds to a region of Rad5 containing a PIP-like motif (residues 6 to 13) [76]. As mentioned above, this is possibly the same motif that Rad5 uses to bind PCNA, although this has not yet been experimentally tested.

#### 4.3.3. Rad18

As described above, Rad5 interacts with Rad18, the E3 ubiquitin ligase that is necessary for translesion synthesis [64]. This interaction has been partially mapped to a region of Rad5 spanning residues 1 to 556, which contains the N-terminal disordered regions and the HIRAN domain. The structural basis and functional implications of this interaction are unclear. However, given that Rad5 binds to unmodified PCNA and to mono-ubiquitylated PCNA with the same affinity [77], the interaction between Rad5 and Rad18 may play a role in recruiting Rad5–Ubc13–Mms2 to mono-ubiquitylated PCNA. 

## 5. The Role of PCNA Modifications in DNA Damage Bypass

The coordination of these DNA damage bypass pathways is not well understood. A common view of the role of Rad6–Rad18 and of Ubc13–Mms2–Rad5 in regulating DNA damage bypass is as follows. When replication forks stall upon encountering DNA damage, Rad6–Rad18 catalyzes the mono-ubiquitylation of PCNA. This signals for the recruitment of non-canonical polymerases such as pol η, Rev1, and pol ζ. If translesion synthesis fails, then Ubc13–Mms2–Rad5 catalyzes the poly-ubiquitylation of PCNA. This signals for fork reversal and template switching.

The protein–protein interactions discussed above and several observations in the literature cast doubt on the traditional model. For example, pol η interacts with the Rad6–Rad18 complex, and this interaction is essential for the recruitment of these proteins to stalled replication forks [63,78,79,80]. Moreover, the presence of pol η increases the efficiency with which Rad6–Rad18 catalyzes the mono-ubiquitylation of PCNA both in vitro and in vivo [80]. This strongly suggests that pol η directly facilitates the transfer of the ubiquitin from the Rad6 protein to PCNA. This likely occurs because pol η stabilizes the complex of Rad6–Rad18 and PCNA in a more catalytically active conformation. Regardless of the specifics of the mechanism, this implies that pol η is already present in the complex with Rad6–Rad18 and with PCNA when the ubiquitin transfer reaction occurs. 

**Figure 5:**
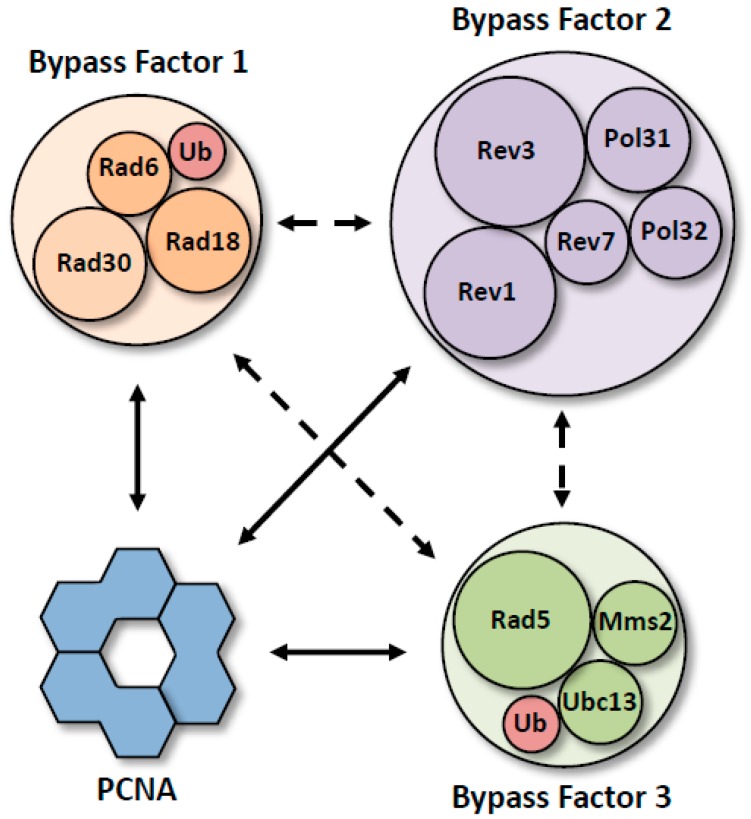
Composition of the putative bypass factors. Diagrams showing the protein components of putative Bypass Factor 1 (orange) containing pol η (Rad30), Bypass Factor 2 (purple) containing pol ζ (Rev3–Rev7–Pol31–Pol32), and Bypass Factor 3 (green) containing Rad5. Ubiquitin moieties are shown in red. Solid arrows represent direct interactions with PCNA that allow for the formation of PCNA tool belts. Dashed arrows represent interactions that allow for the formation of alternative architectures.

These pieces of evidence suggest that the mono-ubiquitylation and poly-ubiquitylation of PCNA do not merely act as signals to recruit the non-canonical polymerases and fork-remodeling helicase. Instead, they suggest that the ubiquitylation machinery plays a more direct role, recruiting these other proteins. According to this alternative view, DNA damage bypass is carried out by the coordinated activity of three multi-protein factors (Figure 5). The first factor, which we dub Bypass Factor 1, is comprised of Rad6–Rad18 and pol η. The second factor, which we refer to as Bypass Factor 2, is comprised of Rev1 and pol ζ. The third factor, which we call Bypass Factor 3, is comprised of Ubc13–Mms2–Rad5. These factors may act sequentially or may assemble into a large complex at a stalled replication fork, a “bypassosome”, comprised of PCNA and the three bypass factors.

Whether or not there is a bypassosome, the evidence reviewed here provides a compelling case for the presence of three bypass factors. According to this model, Bypass Factor 1 is directed to the stalled replication fork via its Rad6–Rad18 components. In this way, its pol η component rides piggyback on its Rad6–Rad18 components. If this model is correct, then pol η is already present at the stalled replication fork when the ubiquitin moiety is transferred to PCNA. 

Next, Bypass Factor 2 is directed to the stalled fork via interactions of its Rev1 component with ubiquitin-modified PCNA or with the pol η component of Bypass Factor 1. Bypass Factor 2 may also be directed to stalled replication forks via interactions of its pol ζ with ubiquitin-modified PCNA.

Finally, Bypass Factor 3 is directed to the stalled replication fork via the E2 ubiquitin-conjugating and E3 ubiquitin ligase activities of Ubc13–Mms2–Rad5. Because Rad5 also possesses a fork-remodeling helicase activity, this activity rides piggyback on the ubiquitylation activity. This factor may also be recruited via interactions of its Rad5 component with mono-ubiquitylated PCNA, with the Rad18 component of Bypass Factor 1, or with the Rev1 component of Bypass Factor 2. Because poly-ubiquitylation of PCNA likely requires the mono-ubiquitylation of PCNA, the recruitment of Bypass Factor 3 likely occurs after the recruitment of Bypass Factor 1.

## 6. Conclusions

In this review, we have discussed the regulation of translesion synthesis and template switching by post-translational modifications of PCNA. We have focused on the structures, functions, and interactions of the E3 ubiquitin ligases Rad18 and Rad5, which facilitate the mono-ubiquitylation and poly-ubiquitylation of PCNA. We have focused on these E3 ubiquitin ligases because they play an important and arguably more direct role in orchestrating the steps of translesion synthesis and template switching than was initially thought. Unfortunately, we still know very little about the structures, functions, and interactions of these proteins.

One reason we know so little about these E3 ubiquitin ligases is that they contain extensive regions of intrinsic disorder conferring to them a high degree of conformational flexibility. Another reason is that they are components of large, dynamic, multi-protein complexes that likely change their composition during the course of the DNA damage bypass process. Consequently, these are extremely difficult proteins to study from a structural and biochemical perspective because traditional experimental approaches, such as X-ray crystallography, NMR, and even cryo-electron microscopy, do not have the capability to tackle such conformationally and compositionally flexible systems. 

Fortunately, new approaches, both experimental and computational, are emerging that should allow us to gain valuable new structural and biochemical information about such conformationally flexible, multi-protein complexes. For example, hybrid approaches, such as full ensemble methods that combine small-angle X-ray scattering data and molecular dynamics simulations, should be extremely useful in studying such systems [81]. Similarly, single-molecule total internal reflection fluorescence microscopy studies of the assembly and composition of multi-protein complexes should also prove useful [82]. Ultimately, these emerging technologies should provide important and exciting insights into these DNA damage bypass pathways and the maintenance of genome stability. 

## Abbreviations

HIRAN, HIP116 Rad5p N-terminal; HLTF, helicase-like transcription factor; PCNA, proliferating cell nuclear antigen; PIP, PCNA-interacting protein; pol, polymerase; R6BD, Rad6-binding domain; RFC, replication factor C; RING, really interesting new gene; RPA, replication protein A; SAP, SAF-A/B, Acinus, Pias; SAXS, small-angle X-ray scattering; SIM, SUMO-interacting motif; UBZ, ubiquitin-binding, zinc-binding.
